# Infection with *Toxocara canis* Inhibits the Production of IgE Antibodies to α-Gal in Humans: Towards a Conceptual Framework of the Hygiene Hypothesis?

**DOI:** 10.3390/vaccines8020167

**Published:** 2020-04-06

**Authors:** Adnan Hodžić, Lourdes Mateos-Hernández, Emilie Fréalle, Patricia Román-Carrasco, Pilar Alberdi, Muriel Pichavant, Veronica Risco-Castillo, Delphine Le Roux, Jérôme Vicogne, Wolfgang Hemmer, Herbert Auer, Ines Swoboda, Georg Gerhard Duscher, José de la Fuente, Alejandro Cabezas-Cruz

**Affiliations:** 1 Institute of Parasitology, Department of Pathobiology, University of Veterinary Medicine Vienna, 1210 Vienna, Austria; 2 UMR BIPAR, INRAE, ANSES, Ecole Nationale Vétérinaire d’Alfort, Université Paris-Est, 94706 Maisons-Alfort, France; lmateoshernandez@hotmail.com (L.M.-H.); delphine.le-roux@vet-alfort.fr (D.L.R.); 3 CNRS, Inserm, CHU Lille, Institut Pasteur de Lille, U1019–UMR 8204–CIIL–Center for Infection and Immunity of Lille, University of Lille, F-59000 Lille, France; emilie.frealle2@univ-lille.fr; 4 CHU Lille, Laboratory of Parasitology and Mycology, F-59000 Lille, France; muriel.pichavant@pasteur-lille.fr; 5 Molecular Biotechnology Section, FH Campus Wien, University of Applied Sciences, 1030 Vienna, Austria; patricia.roman_carrasco@chello.at (P.R.-C.); ines.swoboda@fh-campuswien.ac.at (I.S.); 6 SaBio, Instituto de Investigación en Recursos Cinegéticos (IREC-CSIC-UCLM-JCCM), Ronda de Toledo s/n, 13005 Ciudad Real, Spain; maria.alberdi@uclm.es (P.A.); jose_delafuente@yahoo.com (J.d.l.F.); 7 EA 7380 Dynamyc, UPEC, USC, ANSES, Ecole Nationale Vétérinaire d’Alfort, Université Paris-Est, 94706 Maisons-Alfort, France; veronica.risco-castillo@vet-alfort.fr; 8 CNRS, Inserm, CHU Lille, Institut Pasteur de Lille, U1019–UMR9017–CIIL–Center for Infection and Immunity of Lille, University of Lille, F-59000 Lille, France; jerome.vicogne@ibl.cnrs.fr; 9 FAZ-Floridsdorf Allergy Center, 1210 Vienna, Austria; hemmer@faz.at; 10 Department of Medical Parasitology, Institute of Specific Prophylaxis and Tropical Medicine, Center of Pathophysiology, Infectiology and Immunology, Medical University of Vienna, 1090 Vienna, Austria; herbert.auer@meduniwien.ac.at; 11 AGES-Austrian Agency for Health and Food Safety, 1220 Vienna, Austria; georg.duscher@ages.at; 12 Department of Veterinary Pathobiology, Center for Veterinary Health Sciences, Oklahoma State University, Stillwater, OK 74078, USA

**Keywords:** α-Gal, allergy, hygiene hypothesis, immune response, suppression, *Toxocara canis*

## Abstract

α-Gal syndrome (AGS) is a type of anaphylactic reaction to mammalian meat characterized by an immunoglobulin (Ig)E immune response to the oligosaccharide α-Gal (Galα1-3Galβ1-4GlcNAc-R). Tick bites seems to be a prerequisite for the onset of the allergic disease in humans, but the implication of non-tick parasites in α-Gal sensitization has also been deliberated. In the present study, we therefore evaluated the capacity of helminths (*Toxocara canis*, *Ascaris suum*, *Schistosoma mansoni*), protozoa (*Toxoplasma gondii*), and parasitic fungi (*Aspergillus fumigatus*) to induce an immune response to α-Gal. For this, different developmental stages of the infectious agents were tested for the presence of α-Gal. Next, the potential correlation between immune responses to α-Gal and the parasite infections was investigated by testing sera collected from patients with AGS and those infected with the parasites. Our results showed that *S. mansoni* and *A. fumigatus* produce the terminal α-Gal moieties, but they were not able to induce the production of specific antibodies. By contrast, *T. canis*, *A. suum* and *T. gondii* lack the α-Gal epitope. Furthermore, the patients with *T. canis* infection had significantly decreased anti-α-Gal IgE levels when compared to the healthy controls, suggesting the potential role of this nematode parasite in suppressing the allergic response to the glycan molecule. This rather intriguing observation is discussed in the context of the ‘hygiene hypothesis’. Taken together, our study provides new insights into the relationships between immune responses to α-Gal and parasitic infections. However, further investigations should be undertaken to identify *T. canis* components with potent immunomodulatory properties and to assess their potential to be used in immunotherapy and control of AGS.

## 1. Introduction

The α-Gal epitope (Galα1-3Galβ1-4GlcNAc-R) is an oligosaccharide ubiquitously present on glycoproteins and glycolipids of non-primate mammals, prosimians, and New World monkeys [1]. In these mammals, the glycosylation enzyme α-1,3-galactosyltransferase (α1,3GT) catalyzes the synthesis of the glycan molecule by transferring galactose from uridine diphosphate (UDP)-Gal to N-acetyllactosaminide [2]. By contrast, humans and ancestral anthropoid primates lack the capacity to produce the α-Gal epitope due to the functional disruption of the α1,3GT gene, caused by several deletions in the DNA sequence that encodes premature stop codons [3,4]. This unique evolutionary event resulted in a complete loss of immune tolerance to the α-Gal epitope and the consequent ability of all immunocompetent crown catarrhines to generate a large amount of anti-α-Gal antibodies (Abs) [5,6]. Anti-α-Gal Abs, principally immunoglobulin (Ig)G, IgM, and IgA isotypes, are typically produced by healthy human individuals as an immunological response to continuous antigenic stimulation by gastrointestinal bacteria producing α-Gal on their outer surface [7]. Interestingly, the resulting IgG and IgM Abs were found to be protective against important microbial and parasitic infections [8,9,10,11]. However, hyperproduction of specific IgE Abs to α-Gal triggered by bites of ixodid ticks (Acari: Ixodidae) may lead to a delayed and potentially fatal anaphylactic reaction to mammalian meats (e.g., beef, pork, lamb) and other α-Gal-containing products [12,13]. This recently recognized type of food allergy, also known as α-Gal syndrome (AGS) or red meat allergy, seems to be an emerging global problem with an increasing prevalence reported in all continents [14,15]. In general, affected patients suffer from urticaria, angioedema, abdominal pain, and anaphylaxis that usually occur 3–6 h following the consumption of mammalian food products [12]. Remarkably, most of the patients who developed the allergic reaction had tolerated red meat for many years before being bitten by a tick, which suggests that as-yet-unknown tick salivary antigen(s) break the oral tolerance to food allergens and induce the anaphylactic reaction [16,17].

The association between tick bites and anaphylaxis to mammalian meat was first described in 2007 [18]. Later, Commins et al. [12] identified α-Gal as a source of delayed allergic reactions to mammalian meat in a group of patients from the USA. Further studies have demonstrated the occurrence of terminal α-Gal moieties in saliva of several tick species [19,20,21,22,23,24,25,26] and a more recent publication showed that ticks are able to synthesize α-Gal with functional galactosyltransferases (GTs), which are also involved in tick feeding, tick–pathogen interactions and potentially in the development of AGS [27]. Multiple tick species seem to play a role in α-Gal sensitization in humans worldwide. However, clinical cases of typical AGS, recently described in patients exposed to bites of chiggers, i.e., larval stages of trombiculid mites (Acari: Trombiculidae), may raise the possibility that other, non-tick parasites, may contribute to α-Gal sensitization and the subsequent development of the anaphylactic reaction to mammalian meat [28]. Besides, some pet-associated endoparasites (e.g., *Toxocara* spp., *Echinococcus* spp.) have been proposed to potentially induce sensitization to α-Gal in humans [29,30,31,32]. Indeed, the presence of α-Gal as a capping structure of complex *N*-glycans has been observed in several helminth species such as *Echinococcus granulosus* [33], *Parastrongylus tenuis* [34] and *Haemonchus contortus* [35]. However, the immunological potential of the glycan moieties produced by these parasites remains unknown.

Therefore, the present study aimed to: (i) assess the capacity of zoonotic parasites (*Toxocara canis*, *Ascaris suum*, *Schistosoma mansoni*, *Toxoplasma gondii*) and parasitic fungi (*Aspergillus fumigatus*) to cause α-Gal sensitization following the production of glycotope-specific IgE Abs in infected individuals; (ii) to investigate the possible correlation between immune responses to α-Gal and human infections caused by these pathogenic agents.

## 2. Materials and Methods

### 2.1. Study Population

The population of this retrospective, double-cohort study consisted of subjects from Austria and France. The Austrian cohort included serum samples from 47 individuals of different sex and age (12–73 years old) with a history of allergic reactions after mammalian meat consumption attending the Floridsdorf Allergy Center (FAZ) in Vienna, Austria. All patients reported delayed reactions with urticaria and anaphylaxis as the most dominant symptoms and had serum IgE Abs against α-Gal. The Ab levels to α-Gal-containing commercial allergens (e.g., beef, pork) were assessed by ImmunoCAP assay (Thermo Fisher Scientific, Uppsala, Sweden). The cut-off for allergen-specific IgE was ≥0.35 kU_A_/L. Sera from nine healthy individuals were also included in the study and served as a negative control in serological tests.

The French group consisted of adult patients with chronic toxoplasmosis (n = 14), schistosomiasis (n = 6), hematology patients with invasive aspergillosis (n = 19) and patients with visceral larva migrans (VLM) syndrome caused by *T. canis* (n = 22). Serum samples from healthy (n = 16) and immunosuppressed hematology (n = 18) adult patients from France were used as controls in the present study. In addition, sera from healthy newborns (n = 12) and those with congenital toxoplasmosis (n = 27) were also included in the study. Serological testing for the detection of anti-*Toxocara*, anti-*Schistosoma*, and anti-*T. gondii* antibodies and molecular detection of *T. gondii* were performed according to the recommendations of the French National Authority for Health [36,37,38]. All *Toxocara* and *Schistosoma* infections were confirmed by a positive immunoblot test (LDBio Diagnostics, Lyon, France). Chronic toxoplasmosis corresponded to the positive detection of IgG and the absence of IgM using Enzygnost Toxoplasmose assays (Siemens Healthcare Diagnostics, Saint-Denis, France). For congenital toxoplasmosis, IgM detection in serum was performed using Toxo-ISAGA IgM (BioMérieux, Lyon, France), and a Rep529-targeting PCR was used for detection of *T. gondii* DNA in amniotic fluid or blood samples [39]. For aspergillosis, detection of galactomannan was performed using Platelia^TM^ *Aspergillus* Ag kit (Bio-Rad Laboratories Inc., Marnes-la-Coquette, France), and patients were classified according to the criteria of the European Organization for the Research and Treatment of Cancer/Mycoses Study Group EORTC/MSG [40].

### 2.2. Detection of Specific IgG Antibodies to Toxocara canis, Ascaris suum and Toxoplasma gondii in Sera of Patients with α-Gal Syndrome (AGS)

Sera from the allergic patients were screened for Abs specific to *T. canis*, *A. suum*, and *T. gondii*. Serological tests for *T. canis* included an indirect enzyme-linked immunosorbent assay (Tc-ES-ELISA) based on excretory/secretory antigens (ES) obtained from *T. canis* larvae (Tc-ES) as well as a highly specific immunoblot (IB) test (Tc-ES-IB) first described by de Savigny et al. [41] and Magnaval et al. [42], respectively. Serum samples were considered positive if they yielded a positive reaction in the Tc-ES-ELISA and showed a typical reactivity pattern in the Tc-ES-IB [42].

For detection of specific Abs to *A. suum*, the parasites were collected from the intestines of a naturally infected pig, and the soluble crude extract of a female worm (As-ExAD) was prepared as previously described [43] and used in a modified ELISA test [44]. Briefly, ELISA microplates (Nunc-Immuno^TM^ Plate, Roskilde, Denmark) were coated with 1 μg/mL (100 ng/well) of As-ExAD in carbonate/bicarbonate buffer (0.05 M, pH 9.6). After overnight incubation at 4 °C, the wells were washed five times with phosphate-buffered saline (PBS) containing 0.05% Tween 20 (PBS-T) and then blocked with 1% human serum albumin (HSA; Sigma-Aldrich, St. Louis, MO, USA) in PBS-T for 1 h. After the washing step, the sera were added at a 1:800 dilution in 0.5% HSA/PBS-T to the respective wells and then incubated for 1 h. Goat anti-human IgG (Fc-specific) horseradish-peroxidase (HRP)-conjugated secondary Ab (Sigma-Aldrich, Madrid, Spain) was added at a 1:1000 dilution in 0.5% HSA/PBS-T and incubated for 1 h. All the incubation steps were performed at 37 °C. The reaction was visualized by adding 100 μL ready-to-use 3,3′-5,5′-tetramethylbenzidine (TMB) solution (Thermo Fisher Scientific, Rockford, IL, USA) and then stopped with 50 μL of 0.5 M sulfuric acid. The resulting optical densities (OD) were measured at 450 nm in a microplate ELISA reader (Filter-Max F5, Molecular Devices, San Jose, CA, USA). The samples were considered serologically positive if the OD values were higher than the cut-off point, i.e., the mean OD of nine negative control sera plus four times the standard deviation [43].

A commercially available ELISA test (*Toxoplasma gondii* IgG ELISA, DRG Instruments GmbH, Marburg, Germany) was used to detect *T. gondii*-specific IgG Abs in the serum samples. Analyses were performed according to the manufacturer’s instructions.

### 2.3. Detection of Serum IgG, IgM and IgE Antibodies to α-Gal in Patients Infected with Parasites and Parasitic Fungi

Serum samples collected from the patients with VLM syndrome, schistosomiasis, chronic toxoplasmosis, and aspergillosis were also screened for anti-α-Gal IgG, IgM, and IgE Abs by indirect ELISA following the procedure previously described [22]. In brief, Galα1-3Galβ1-4GlcNAc coupled to HSA (Carbosynth Ltd., Hampshire, UK) was used as an antigen at a concentration of 1 μg/mL (100 ng/well). Sera from the patients (diluted 1:50 in 0.5% HSA/PBS-T) and peroxidase-conjugated goat anti-human IgG (Fc-specific), IgM (µ-chain specific), or IgE (ε-chain specific) (Sigma-Aldrich, Madrid, Spain) at a dilution of 1:1000 were used as primary and secondary Abs, respectively. Anti-α-Gal Ab levels of the parasite-infected patients were compared to those of healthy and immunocompromised controls.

### 2.4. Determination of Antibody Levels to Ixodes ricinus Salivary Gland Proteins (SGP)

Salivary gland proteins (SGP) from unfed adult female *Ixodes ricinus* ticks were extracted as previously described by Hodžić et al. [26]. To assess the previous exposure to tick bites, an indirect ELISA was performed to determine the levels of IgG and IgM Abs to *I. ricinus* SGP in sera from patients with AGS following the protocol published elsewhere [22]. Briefly, 96-well ELISA plates (Nunc-Immuno^TM^ Plate, Roskilde, Denmark) were coated with 0.5 μg/mL (50 ng/well) of soluble *I. ricinus* SGP diluted in carbonate/bicarbonate buffer (0.05 M, pH 9.6) and incubated overnight at 4 °C. Following the washing step, the plates were blocked with 1% HSA/PBS-T, incubated for 1 h at 37 °C, and then washed again with PBS-T. Subsequently, sera diluted 1:100 in 0.5% HSA/PBS-T were added to the wells and incubated for 1 h at 37 °C. HRP-conjugated goat anti-human IgG (Fc-specific) and IgM (µ-chain specific) (Sigma-Aldrich, Madrid, Spain) at a 1:1000 dilution were used as secondary Abs. The following steps were performed as described above. The cut-off was determined as two times the mean OD value of the blank controls [45].

### 2.5. Detection of α-Gal Epitopes in Toxocara canis, Ascaris suum, Schistosoma mansoni, Toxoplasma gondii and Aspergillus fumigatus

#### 2.5.1. Preparation of the Organisms for Immunoblot, Indirect ELISA, Immunofluorescence and Flow Cytometry

Adult *T. canis* worms were collected from a naturally infected puppy after deworming, whereas *A. suum* worms were obtained from the intestines of slaughtered pigs. Larval ES antigens of *T. canis* (Tc-ES) were harvested from in vitro culture as described before [46]. The soluble whole-worm antigens (Tc-ExAD, As-ExAD) were prepared following the previously described procedure [43] and used in Western blot and ELISA [35] analysis. *Toxocara canis* larval ES products (Tc-ES) were used as a native antigen in both assays.

A Puerto Rican strain of *S. mansoni* was maintained in the laboratory using the snail (*Biomphalaria glabrata*) as its intermediate host, and the golden hamster (*Mesocricetus auratus*) as a definitive host. Cercariae were collected after being released from infected snails as previously reported [47].

To obtain *T. gondii* tachyzoites, human foreskin fibroblast (HFF) cells (kindly provided by Dr Morisse, Université de Tours, France) were maintained at 37 °C and 5% CO_2_ in Dulbecco’s Modified Eagle Medium (DMEM) supplemented with 10% fetal calf serum, 4 mM glutamine, 50 U/mL penicillin and 50 µg/mL streptomycin (all from Gibco, Thermo Fisher Scientific, Illkirch, France). Cells were infected three days before harvesting with 100 tachyzoites of the PRU strain, expressing yellow fluorescent protein (YFP) (kindly provided by Dominique Soldati-Favre, University of Geneva, Switzerland).

The clinical strain CBS144.89 of *A. fumigatus* was used in the study. All mycological cultures were performed on Sabouraud-Dextrose (SD) agar plates supplemented with chloramphenicol (50 mg/L), incubated at 37 °C and sub-cultured twice a week. To prepare the conidia, *A. fumigatus* was grown for 2–3 days at 37 °C. Conidia were subsequently harvested by resuspending in PBS containing 0.01% (vol/vol) Tween 20 (PBST), filtered in a 70 µm diameter nylon cell strainer (ClearLine^®^ Dominique Dutscher, Brumath, France), washed by centrifugation at 3500× *g* for 10 min, resuspended in PBST and then counted using a Malassez counting chamber.

After preparation, *T. gondii* tachyzoites, *S. mansoni* cercariae, and *A. fumigatus* conidia were used to detect the expression of terminal α-Gal residues in immunofluorescence and flow cytometry.

#### 2.5.2. Western Blot

Briefly, 15 µg of the adult worm crude extracts (Tc-ExAD, As-ExAD) and Tc-ES antigens were resolved by sodium dodecyl sulfate-polyacrylamide gel electrophoresis (SDS-PAGE) following the protocol [35]. After electrophoresis, the resolved proteins were transferred to nitrocellulose membranes (Trans-Blot-Turbo-Transfer-System, Bio-Rad Laboratories Inc., Feldkirchen, Germany). The membranes were blocked with 1× casein solution (Vector Laboratories Inc., Burlingame, CA, USA) and incubated with a monoclonal mouse anti-α-Gal antibody (mAb) M86 (Enzo LifeScience Inc., Farmingdale, NY, USA) diluted 1:5 in Tris buffered saline buffer (TTBS; 100 mM Tris, 0.9% NaCl, 0.1% Tween 20). After rinsing in TTBS buffer for 15 min (3 × 5 min), blots were incubated with HRP-goat anti-mouse IgM (Bio-Rad Laboratories Inc., Feldkirchen, Germany), diluted 1:300 in TTBS. All the incubation steps were carried out at room temperature. Immunoreactive proteins were detected by using the DAB-peroxidase substrate kit (Vector Laboratories Inc., Burlingame, CA, USA) and following the manufacturer’s instructions. Galα1-3Gal-HSA antigen (5 µg; Dextra Laboratories, Reading, UK) was used as a positive control.

#### 2.5.3. Indirect ELISA

For the detection of α-Gal in the glycoproteins of *T. canis* (Tc-ExAD), *A. suum* (As-ExAD) and the larval ES products of *T. canis* (Tc-ES), the parasites’ soluble antigens (1 μg/mL or 100 ng/well) were coated overnight at 4 °C on a microplate (Nunc-Immuno^TM^ Plate, Roskilde, Denmark). Galα1-3Gal-HSA antigen (Dextra Laboratories, Reading, UK) served as a positive control (0.5 μg/mL or 50 ng/well). After blocking with 1% HSA (1 h/37 °C) and washing with PBS-T, the presence of α-Gal epitopes in the protein extracts was evaluated by the M86 mAb (Enzo LifeScience Inc., Farmingdale, NY, USA) diluted at 1:400. HRP-goat anti-mouse IgM (Bio-Rad Laboratories Inc., Feldkirchen, Germany) was used at a 1:4000 dilution as a secondary Ab, and the following ELISA steps were performed as described above.

#### 2.5.4. Immunofluorescence and Flow Cytometry

Uninfected and *Toxoplasma* infected cells were fixed with 4% paraformaldehyde and incubated with 3% HSA (Sigma-Aldrich, St. Louis, MO, USA) in PBS for 1 h at room temperature. After subsequent incubation with the monoclonal Ab M86 (Enzo LifeScience Inc., Farmingdale, NY, USA) diluted 1:50 in 3% HSA/PBS for 14 h at 4 °C, the secondary antibody Alexa Fluor^®^ 647 (red) goat anti-mouse IgM (Abcam, Cambridge, UK) was used at 2 µg/mL in 3% HSA/PBS for 1 h at room temperature. Stained samples were mounted in ProLong Antifade reagent containing DAPI (Molecular Probes, Eugene, OR, USA). The cell preparations were examined using a Zeiss LSM 800 laser scanning confocal microscope (Carl Zeiss, Oberkochen, Germany) with oil immersion objectives (×63).

Identification and relative expression of α-Gal epitopes in *S. mansoni* cercariae and *A. fumigatus* conidia was performed by immunofluorescence and flow cytometry following the previously described procedures [27]. The α-Gal-specific monoclonal antibody M86 (Enzo LifeScience Inc., Farmingdale, NY, USA) and FITC-labelled goat anti-mouse IgM (Abcam, Cambridge, UK) were used as primary and secondary antibodies, respectively.

### 2.6. Statistical Analyses

All data were statistically analyzed using GraphPad 5 Prism software (GraphPad Software Inc., San Diego, CA, USA). One-way analysis of variance (ANOVA) with Dunnett’s multiple comparison posthoc test was applied for individual comparisons of Ab levels to the control groups, while the Kruskal–Wallis test with Dunn’s posthoc test was used to compare the differences between infected groups. The differences in the reactivity of the Abs to As-ExAD and tick SGP were tested using the Mann–Whitney *U* test. Differences were considered significant when *p* < 0.05. The confidence interval (95% CI) for the proportions was calculated by the Wilson score method [48].

### 2.7. Ethics Statement

All healthy blood donors and patients consented to participate in the study (this also applies to parents of infants). The study was approved by the Ethics Committees of the cities of Vienna (EK-12-126-0712) and Lille (referral number: 2014-45) and conformed to the principles of the Declaration of Helsinki. All methods were performed following the relevant guidelines and regulations.

## 3. Results

### 3.1. Expression of Terminal α-Gal Moieties on Glycoproteins and the Surface of the Tested Helminths, Protozoa and Parasitic Fungi

**Figure 1:**
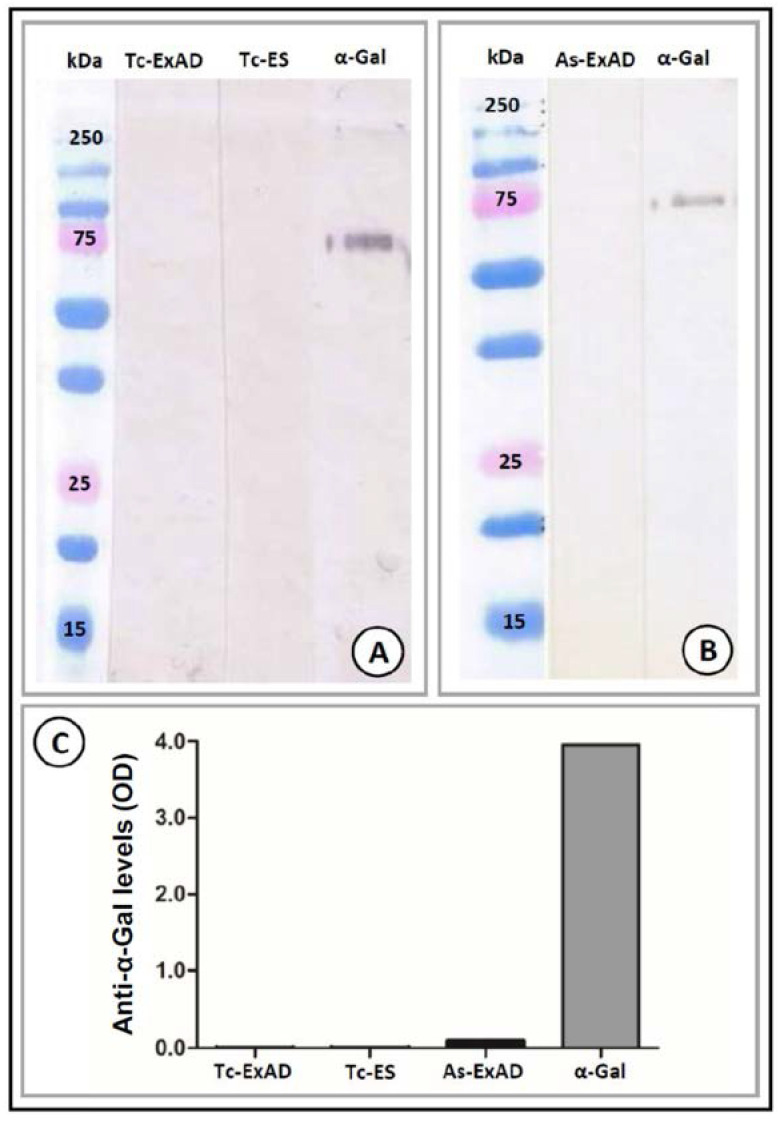
Detection of α-Gal on glycoproteins of *Toxocara canis* and *Ascaris suum* by Western blot ((**A**,**B**), respectively) and indirect ELISA (**C**). Disaccharide Galα1-3Gal-HSA served as a positive control in both assays. The presence of α-Gal in the soluble proteins was assessed by a highly specific monoclonal anti-α-Gal antibody M86. Abbreviations: Tc-ExAD—crude extract of an adult female *T. canis* worm; Tc-ES—larval excretory/secretory antigens of *T. canis* (native antigen); As-ExAD—crude extract of an adult female *A. suum* worm; α-Gal—Galα1-3Gal-HSA antigen; OD—optical density.

To assess the potential of the tested organisms to induce an immune response to α-Gal in humans, we investigated the presence of α-Gal epitopes on glycoproteins of adult worms and larval ES products, as well as on the surface of the developmental stages of helminth and protozoan parasites and parasitic fungi. α-Gal production was detected using the highly specific mAb M86 that recognizes synthetic and naturally produced terminal α-Gal epitopes, in particular, the α1-3 linkage (but not the α1-4 or β-Gal linkages) on different glycoproteins and glycolipids [49]. The synthetic Galα1-3Gal-HSA epitope could be detected with the M86 mAb in both Western blot (the band at the expected molecular weight of 72 kDa) and ELISA, but no detectable binding to glycoproteins derived from adult *T. canis* (Tc-ExAD) and *A. suum* (As-ExAD) worms or ES antigens of *T. canis* larvae (Tc-ES) was observed (Figure 1).

**Figure 2:**
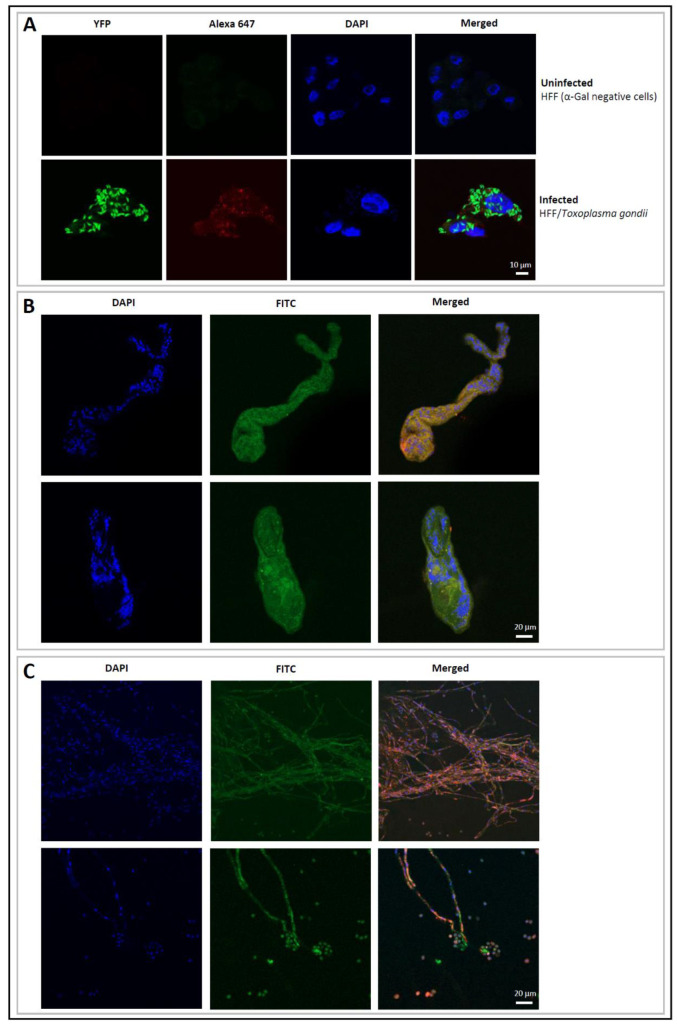
Expression of the terminal α-Gal moieties on the surface of *Toxoplasma gondii* tachyzoites (**A**); *Schistosoma mansoni* cercariae (**B**) and *Aspergillus fumigatus* conidia (**C**) assessed by immunofluorescence. Uninfected human foreskin fibroblast (HFF) cells, which do not express α-Gal, served as a negative control (**A**). The α-Gal-specific monoclonal antibody M86 (primary antibody) and the Alexa Fluor^®^ 647 goat anti-mouse IgM (secondary antibody) were used to detect the production of α-Gal in *Toxoplsma gondii* tachyzoites expressing yellow fluorescent protein (YFP) (**A**). Goat anti-mouse IgM-FITC was used as secondary antibody for detection of α-Gal (green) in *Schistosoma manosni* (**B**) and *Aspergillus fumigatus* (**C**). Cell nuclei were stained with DAPI (blue) (**A**–**C**).

This observation is suggestive of the absence of glycoproteins bearing terminal α-galactosylated moieties in these parasites and their products. The α-Gal glycotope was not detected on infective *T. gondii* tachyzoites (Figure 2A) by immunofluorescence, but its presence was observed on the surface of *S. mansoni* cercariae (Figure 2B) and conidia of *A. fumigatus* (Figure 2C). The M86 mAb specifically binds to the surface membranes of the organisms, but not to their nuclei (Figure 2).

**Figure 3:**
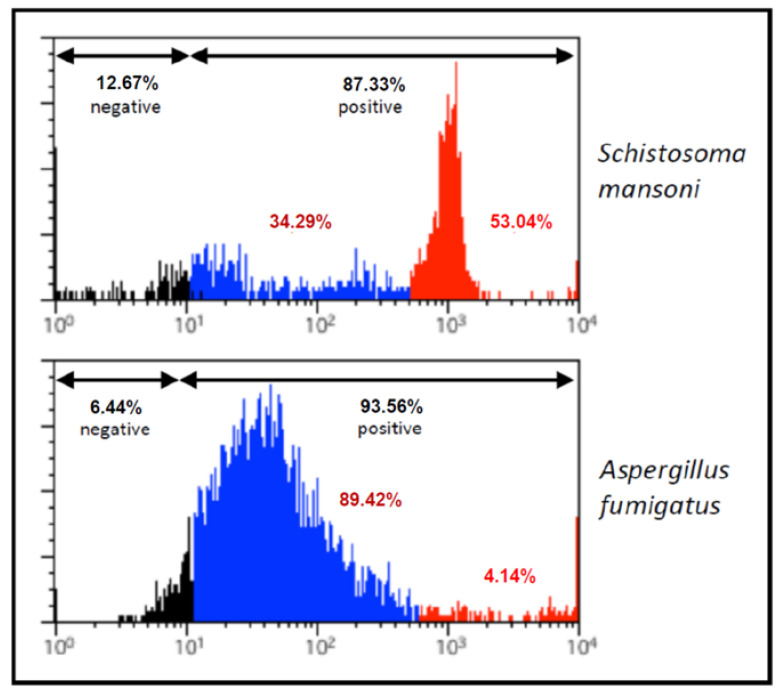
Flow cytometry analysis of *Schistosoma mansoni* cercariae and *Aspergillus fumigatus* conidia counts after incubation with the α-Gal-specific monoclonal antibody M86 (primary antibody) and FITC-conjugated goat anti-mouse IgM (secondary antibody). The organisms are represented in a histogram to evaluate the relative expression of α-Gal. Unstained cells served as a negative control (black). Different proportions with low (blue) and high (red) levels of α-Gal were observed in both agents.

The strong binding of the mAb M86 to α-Gal epitopes in *S. mansoni* and *A. fumigatus* was further confirmed by flow cytometry, where different proportions of α-Gal levels were recorded in the populations of both organisms tested (Figure 3).

These results provide strong evidence that *S. mansoni* and *A. fumigatus* abundantly express α-Gal on their surfaces, which makes the epitope highly accessible to the host immune system and gives rise to the possibility that these pathogenic agents could be incriminated in α-Gal sensitization.

### 3.2. Association between Immune Responses to α-Gal and Exposure to the Infectious Agents

**Figure 4:**
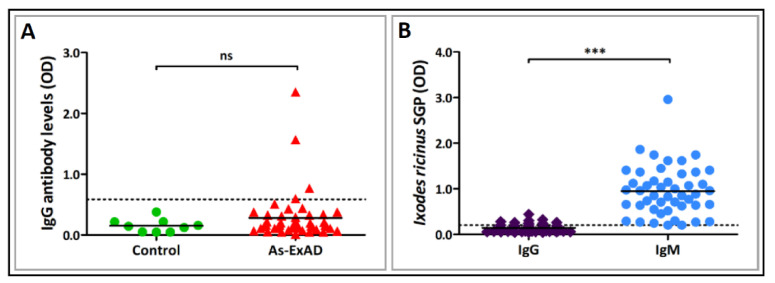
Reactivity of serum antibodies to *Ascaris suum* and *Ixodes ricinus* ticks in patients with α-Gal syndrome. (**A**) IgG antibodies to crude extract derived from an adult female *Ascaris suum* worm (As-ExAD). Mann–Whitney *U* test: ns—not significant. (**B**) Levels of specific IgG and IgM antibodies to *Ixodes ricinus* salivary gland proteins (SGP). Mann–Whitney *U* test: *** *p* < 0.0001.

To investigate the immunogenic properties of α-Gal expressed on the surface of *S. mansoni*, *T. gondii*, and *A. fumigatus* and the possible association between immune responses to α-Gal and exposure to different parasitic organisms, we tested sera from two groups of patients (Table 1). The first group included individuals with AGS and increased levels of specific anti-α-Gal IgE Abs (median: 7.5 kU_A_/L, mean: 29.3 kU_A_/L, range: 0.39 to ≥100 kU_A_/L). Serum samples from these patients were additionally tested for IgG Abs generated towards three common zoonotic parasites for which humans serve as paratenic (*T. canis*, *A. suum*) or intermediate hosts (*T. gondii*). Among 47 samples tested, serum Abs to *T. canis* were detected in only three (6.4%; 95% CI: 2.2–17.1%) by both Tc-ES-ELISA and Tc-ES-IB tests (data not shown). Remarkably, the same three subjects were reactive to As-ExAD crude antigens derived from an adult female *A. suum* (Figure 4A) and had the lowest concentration of IgE Abs to α-Gal among the patients tested (0.39 kU_A_/L, 0.43 kU_A_/L and 0.51 kU_A_/L). Sera from the healthy control group did not show reactivity (Figure 4A), and there was no statistically significant difference in specific IgG production (*p* > 0.05) between the allergic individuals and the control group (Figure 4A). None of the tested patients had detectable IgG Abs against *T. gondii*.

In order to evaluate the previous exposure of the allergic patients to tick bites and the possible implication of *I. ricinus* in the development of the anaphylactic reaction to mammalian meats, we measured IgG and IgM Ab levels against SGP isolated from female *I. ricinus* ticks. While only a few individuals developed IgG to *I. ricinus* SGP (12.8%; 95% CI: 6.0–25.2%), all of them had increased anti-SGP IgM Abs (100.0%; 95% CI: 92.4–100.0%), as observed by indirect ELISA (Figure 4B). The amounts of IgM Abs produced to tick SGP were significantly higher (*p* < 0.0001) compared to those of IgG (Figure 4B).

**Figure 5:**
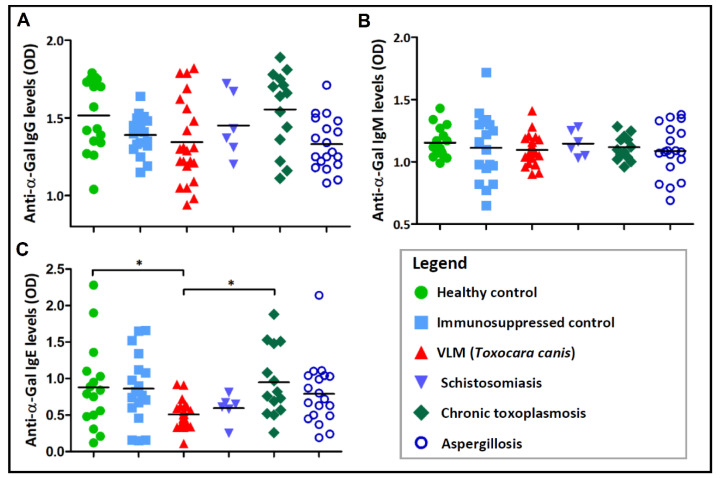
Reactivity of (**A**) IgG, (**B**) IgM, and (**C**) IgE antibodies to α-Gal in patients with schistosomiasis, chronic toxoplasmosis, aspergillosis, and visceral larva migrans (VLM) caused by *Toxocara canis* as measured by indirect ELISA. Sera from healthy and immunosuppressed individuals were included as controls. One-way ANOVA with Dunnett’s multiple comparison test was applied for individual comparisons of antibody levels to the control groups, and the Kruskal–Wallis test with Dunn’s posthoc test was used to test the differences between the infected groups. Only statistically significant differences are indicated: * *p* < 0.05.

**Figure 6:**
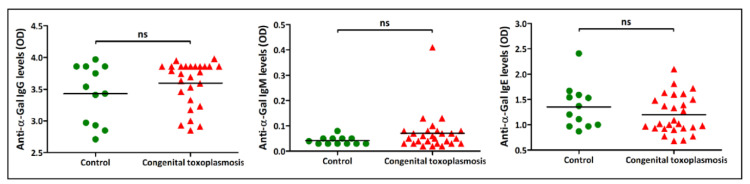
Reactivity of IgG, IgM, and IgE antibodies to α-Gal in newborns with congenital toxoplasmosis as assessed by indirect ELISA. Mann–Whitney *U* test: ns—not significant.

The second group consisted of adult patients with chronic toxoplasmosis, aspergillosis, schistosomiasis and VLM syndrome caused by *T. canis*, and infants with congenital toxoplasmosis (Table 1). Sera obtained from this group of patients and those of healthy and immunosuppressed controls were also tested for IgG, IgM, and IgE Abs to α-Gal (Figure 5 and Figure 6). In adult subjects, levels of anti-α-Gal IgG and IgM were almost equally distributed in all pathogen-infected groups and did not differ statistically (*p* > 0.05) from those observed in the control groups (Figure 5A,B). However, IgE Abs were significantly lower in individuals that tested positive for *T. canis* in comparison to the healthy control group (*p* < 0.05) or the group of patients with chronic toxoplasmosis (*p* < 0.05) (Figure 5C). Differences in IgE levels between other groups were not significant (*p* > 0.05; Figure 5C).

On the other hand, the amounts of all three anti-α-Gal isotypes tested in neonates with congenital toxoplasmosis were not significantly different from those of the healthy control (*p* > 0.05; Figure 6).

Interestingly, healthy infants, as well as those with congenital toxoplasmosis, had higher IgG levels to α-Gal (Figure 6) compared to adults from the control groups or those affected with chronic toxoplasmosis (Figure 5A) (Kruskal–Wallis *U* test: *p* < 0.0001). However, they had an almost undetectable amount of IgM anti-α-Gal Abs (Figure 6), while levels of specific IgE were similar in infants (Figure 6) and adult subjects (Figure 5C) of both healthy and *T. gondii*-infected groups (Kruskal–Wallis *U* test: *p* > 0.05).

## 4. Discussion

Elevated levels of IgE Abs specific to α-Gal, but not associated with AGS, observed in a large proportion of individuals with chronic parasitic infections from rural and urban areas in Kenya and Zimbabwe, certainly raises the possibility that helminths may serve as a source of α-Gal sensitization [29,30,31]. However, the cause of sensitization is not very clear, and no direct link has been made between the immune response to α-Gal and specific parasite species in those research studies. Therefore, detection of the α-Gal epitope in *T. canis*, *A. suum*, *S. mansoni*, and *T. gondii* parasites and *A. fumigatus* fungi was performed in the present study to assess the potential of these infectious agents in promoting α-Gal-specific immune responses in infected humans. Our results revealed high levels of the terminal α-Gal moieties expressed on the surface of *S. mansoni* cercariae and *A. fumigatus* conidia, but not on *T. gondii* tachyzoites. Still, the epitope was not detectable on glycoproteins of the adult worms of *T. canis* (Tc-ExAD) and *A. suum* (As-ExAD) and ES antigens of *T. canis* larvae (Tc-ES). However, van Stijn et al. [35] observed the strong binding of *Griffonia* (*Bandeirea*) *simplicifolia* I isolectin B4 (GSI-B4) to glycoproteins derived from adults of *T. canis*, but the soluble protein extracts did not show reactivity with the anti-α-Gal mAb (M86) in immunoblots and ELISAs. The discrepancy in the results can be explained by the fact that GSI-B4 lectin binds specifically to both α1-3 and α1-4 linkages to α-Gal [50], suggesting that α-Gal within *T. canis* glycoproteins does not occur in an α1-3 linkage [35]. In the same study, the presence of α-Gal could not be confirmed in glycoproteins isolated from adults and cercariae of *S. mansoni* [35]. These results, along with our finding of the epitope on the parasite’s surface, may suggest the possibility that α-Gal is actually bound to an outer membrane lipid moiety rather than to a protein linker. Taken together, the production of α-Gal epitopes in parasites is apparently a species-specific trait, which may play an important role in the host–parasite interactions. The expression of host-like glycan determinants by parasites, a concept known as molecular or glycan mimicry, likely enable them to evade the host immune response [1,34,35]. However, the capacity of parasite-derived glycoproteins and glycolipids containing the terminal α-Gal moieties to initiate immune responses to α-Gal in humans, including a specific IgE response, is yet unknown.

Therefore, to investigate the possible correlation between immune responses to α-Gal and exposure to parasites, we first examined sera from patients with AGS for parasite-specific Abs. Only three individuals from this cohort had detectable Abs against *T. canis* (Tc-EcAD) and the same sera bound to somatic *A. suum* antigens (As-ExAD). In general, high levels of cross-reactivity between *T. canis* and *A. suum* has often been seen in ascarid infections due to the antigenic similarity between these two closely related species that cause human VLM syndrome [43,51]. Considering this and the fact that *T. canis* infection was confirmed by the highly specific Tc-ES-IB assay [42,51], these three patients were likely infected with *T. canis* and not with *A. suum*. By all means, our results demonstrate the absence of any apparent correlation between the parasite infections and AGS. However, α-Gal IgE positivity was strongly associated with a history of recent tick bites, since all patients with the anaphylactic reaction to mammalian meat produced IgM Abs directed against *I. ricinus* SGP. Exposure to *I. ricinus* ticks has often been discussed in terms of AGS development in Europe [24,52,53,54], but the possible contribution of other tick species should not be excluded [22]. To further assess the potential role of parasites and fungi in promoting α-Gal-specific immune responses, sera from the parasite-infected cohort were tested for reactivity to the α-Gal epitope, and specific Ab levels were compared to those of healthy and immunosuppressed controls. Apart from anti-α-Gal IgE levels, which were significantly lower in patients with VLM caused by *T. canis* than in healthy controls or the group with chronic toxoplasmosis, distribution of other Abs between different groups was about the same level. These results support the previous ones observed in patients with AGS and show that the infectious agents, including those expressing α-Gal, are not able to elicit the production of anti-α-Gal Abs in infected humans. However, an interesting observation is that *T. canis* decreased the levels of IgE to α-Gal, suggesting the potential role of the parasite in suppressing the allergic response, but this premise will be discussed later. Furthermore, our results revealed a substantial amount of IgG Abs to α-Gal in infants, which indicates placental transfer of maternal IgG Abs that may protect the infant at a very early age, but seemingly not against congenital toxoplasmosis.

As already seen, *S. mansoni* (cercariae) and *A. fumigatus* (conidia) express the high levels of α-Gal on their outer membranes, but even though the epitope is exposed to the host immune system, these organisms are not capable of triggering a response to α-Gal in the infected subjects. This brings up the question of why does α-Gal from tick saliva or gut microbiota initiate a specific immune response in humans, while the epitope produced by the parasites is not immunogenic? In general, the exact mechanisms behind the production of anti-α-Gal Abs is not well known, and it is still a matter of debate [13,14,55], but there are several possible explanations for this rather intriguing observation. The first hypothesis suggests that α-Gal from tick salivary glycoproteins is presented to antigen-presenting cells (APCs) and B lymphocytes in the context of T helper (Th) 2 cell-mediated immunity induced by tick saliva, which would, in turn, lead to the differentiation of α-Gal-specific B cells into IgE secreting plasma cells [55]. Nonetheless, Portillo et al. [11] hypothesized that different presentation of *N*- and *O*-linked glycopeptides containing α-Gal by activated B cells via major histocompatibility complex class II (MHC-II) to CD4^+^ T cells could induce distinct isotype switching (IgM to IgG or IgM to IgE). The way that α-Gal antigen molecules are captured, processed and presented to CD4^+^ T cells or other unconventional T cell subsets appears to determine whether the antigen is recognized by the host immune system as harmless, like possibly in case of the parasites, or dangerous [14,56]. Another putative mechanism proposes that tick saliva contains factors, such as salivary prostaglandin E2 (PGE2), that could induce class switch recombination on B cells leading to IgE production [13,55,57]. The interaction between α-Gal from tick saliva and the host immune system apparently requires other salivary components (possibly acting as adjuvants) to promote an anti-α-Gal response and high-level sensitization, which may be lacking in parasites and their products [13,56].

A unique evolutionary dialogue that helminths have developed with their hosts, which has led to a protective mechanism that ensures their life-long persistence and survival of both parasite and host, has been discussed [58]. Similar to allergies, helminth infections induce a Th2 immune response associated with cytokines such as interleukin (IL)-4, IL-5, IL-9, IL-13, and increased levels of eosinophils, basophils, mast cells and circulating IgE Abs [58,59]. However, some helminths are able to modulate the host’s immune system by secretion of immunoregulatory molecules which interact with different host cells, resulting in a shift of the typical inflammatory Th2-mediated type towards an anti-inflammatory immune response [60]. This modified Th2-like immune response includes CD4^+^ and CD8^+^ regulatory T (Treg) cells, regulatory B (Breg) cells, alternatively activated macrophages (AAMs) the immunoregulatory cytokines (predominantly IL-10 and transforming growth factor (TGF)-β, as well as IgG4 Abs that counteract proallergic/inflammatory IgE) [60,61]. The induction of the immunosuppressive network and the modification of the T cell profile by helminth infections seems to be essential for parasite survival and may also contribute to the protection and control of other immune-mediated disorders [62,63]. Infections with immunomodulatory helminth species have thus been used to rebalance the altered immune system in individuals suffering from chronic inflammatory and autoimmune diseases [64,65]. The helminth-induced regulatory processes underline the ‘hygiene hypothesis’, which states that exposure to immunomodulatory helminth infections may prevent allergies or autoimmune diseases, while a reduction in environmental exposure to microbes and parasites results in an altered programming of the host immune system and an increased incidence of inflammatory diseases [66,67]. Numerous epidemiological studies have shown an inverse relationship between parasite infections and atopy or other allergic diatheses, supporting the hypothesis [68,69,70]. However, there is discordant evidence that some helminth infections even promote allergic reactions [71,72,73]. For instance, infection with *T. canis* is considered as a risk factor for the development of allergic rhinitis and asthma [74,75]. To our knowledge, there is only one study reporting the relationship between *T. canis* infection and α-Gal IgE sensitization [32]. In that study, sera from 444 adult individuals from the general population in Spain were tested, and the results revealed no association between exposure to *T. canis* and α-Gal IgE positivity. Therefore, we speculate that *T. canis* infection could modulate the host immune system and suppress the production of anti-α-Gal IgE Abs in humans. This hypothesis is mainly based on the fact that IgE levels to α-Gal in our study were significantly lower in the *T. canis*-infected group compared to the control. However, three patients with VLM have developed AGS, but the levels of anti-α-Gal IgE were slightly above the cut-off point and lower than in other patients. The drop in IgE production in the infected group could be mediated by parasite-induced IL-10. This anti-inflammatory cytokine can regulate T cell and B cell activation, increase the IgE-induced activation threshold of basophils, promote an isotype switch from IgE toward non-inflammatory IgG4, and induce IgG4 production by B cells [76]. An increased concentration of IgG4 Abs, IL-10 and other regulatory cytokines induced by helminth infections seem to play an important role in the suppression of allergic diseases in humans [77,78]. *Toxocara canis* is one of the most prevalent parasites affecting dogs and other canids worldwide. In humans and other paratenic hosts (e.g., mice), *T. canis* larvae migrate through different organs and tissues, causing a clinical condition known as VLM syndrome. The larvae release a substantial amount of immunomodulatory ES products, which help them to evade the host immune system and survive in the host tissues for years [79]. Stimulation of mouse splenocytes with *T. canis* ES products resulted in the secretion of regulatory IL-4, IL-5, IL-6, IL-10, and TGF-β cytokines and downregulation of proinflammatory tumor necrosis factor (TNF)-α [80]. In line with this study, the production of IL-10 and TGF-β by macrophages was two times higher in *T. canis*-infected mice compared to an uninfected group, while IL-12 and TNF-α production was significantly suppressed in the infected group [81]. The infection of mice with *T. canis* is apparently associated with alternatively activated macrophages (AAMs), which have anti-inflammatory functions [81]. Furthermore, the expansion of CD4^+^ FoxP3^+^ T cells in lymphoid organs of mice with chronic *T. canis* infection, along with the higher levels of IL-10 in spleen and sera, support their role in the host systemic regulatory response to the infection [82]. The occurrence of Treg cells was observed in both the spleen and peripheral lymph nodes, where CD4^+^ FoxP3^+^, as well as CD8^+^ FoxP3^+^ cells, were increased [82]. The anti-inflammatory role of Tregs has been confirmed in experimental infections with *Trichinella spiralis*, where suppression of gut inflammation was related to the increased expression of IL-10 and TGF-β and expansion of Tregs [83]. Similarly, experimental infection with *A. suum* suppressed lipopolysaccharide (LPS)-induced inflammation in mice through mechanisms mediated by Tregs, regulatory IL-10 and TGF-β [84].

Taken together, *T. canis* infection can induce a strong systemic regulatory Th2 immune response characterized by the secretion of regulatory factors and decreased production of proinflammatory cytokines, which may contribute to the suppression of α-Gal IgE production in humans with AGS. Even unproven, this premise is very appealing, and it merits further investigations and experimental confirmation so that it may help in the prevention and control of AGS. Therefore, future studies should focus on the identification of *T. canis* molecules with potent immunomodulatory properties which could be used in immunotherapy of the allergy. Our findings also add to a better understanding of the relationships between immune responses to α-Gal and parasitic infections. While the parasites tested in our study have no ability to cause α-Gal sensitization in humans, bites of *I. ricinus* seems to be the primary underlying factor inducing the production of IgE Abs to α-Gal in Europe.

## Figures and Tables

**Table 1:** The number of tested and reactive sera collected from patients with α-Gal syndrome (Austria) and those infected with parasites and fungi (France). Expression of terminal α-Gal moieties in targeted organisms as observed by immunoblot, immunofluorescence, and flow cytometry.

Country	AGS	VLM (*Toxocara canis*)	VLM (*Ascaris suum*)	Schistosomiasis	Toxoplasmosis		Aspergillosis	AGS vs. Infection
					Congenital	Chronic		
**Austria** *	47	3	0	n. t.	n/a	0	n. t.	47/3
**France** **	0	22	n. t.	6	27	14	19	0/88
**α-Gal** **epitopes**		*Toxocara canis*	*Ascaris suum*	*Schistosoma* *mansoni*	*Toxoplasma gondii*		*Aspergillus* *fumigatus*	
		no	no	yes	no		yes	

* Sera from nine healthy individuals were included in the study and used as a negative control in the serological tests. ** Serum samples from healthy (n = 16) and immunosuppressed (n = 18) adult patients and healthy newborns (n = 12) were used as controls. AGS—α-Gal syndrome; VLM—visceral larva migrans syndrome (causative agent); n. t. —not tested; n/a—not applicable.
